# Inflammatory Cascades Driven by Tumor Necrosis Factor-Alpha Play a Major Role in the Progression of Acute Liver Failure and Its Neurological Complications

**DOI:** 10.1371/journal.pone.0049670

**Published:** 2012-11-15

**Authors:** Anne Chastre, Mireille Bélanger, Elizabeth Beauchesne, Bich N. Nguyen, Paul Desjardins, Roger F. Butterworth

**Affiliations:** 1 Neuroscience Research Unit, Hôpital Saint-Luc, CRCHUM, Montreal, Canada; 2 Département de pathologie, Hôpital Saint-Luc, CHUM, Montreal, Canada; Virginia Tech, United States of America

## Abstract

**Background/aims:**

Acute liver failure (ALF) due to ischemic or toxic liver injury is a clinical condition that results from massive loss of hepatocytes and may lead to hepatic encephalopathy (HE), a serious neuropsychiatric complication. Although increased expression of tumor necrosis factor-alpha (TNF-α) in liver, plasma and brain has been observed, conflicting results exist concerning its roles in drug-induced liver injury and on the progression of HE. The present study aimed to investigate the therapeutic value of etanercept, a TNF-α neutralizing molecule, on the progression of liver injury and HE in mice with ALF resulting from azoxymethane (AOM) hepatotoxicity.

**Methods/Principal Findings:**

Mice were administered saline or etanercept (10 mg/kg; i.p.) 30 minutes prior to, or up to 6 h after AOM. Etanercept-treated ALF mice were sacrificed in parallel with vehicle-treated comatose ALF mice and controls. AOM induced severe hepatic necrosis, leading to HE, and etanercept administered prior or up to 3 h after AOM significantly delayed the onset of coma stages of HE. Etanercept pretreatment attenuated AOM-induced liver injury, as assessed by histological examination, plasma ammonia and transaminase levels, and by hepatic glutathione content. Peripheral inflammation was significantly reduced by etanercept as shown by decreased plasma IL-6 (4.1-fold; p<0.001) and CD40L levels (3.7-fold; p<0.001) compared to saline-treated ALF mice. Etanercept also decreased IL-6 levels in brain (1.2-fold; p<0.05), attenuated microglial activation (assessed by OX-42 immunoreactivity), and increased brain glutathione concentrations.

**Conclusions:**

These results indicate that systemic sequestration of TNF-α attenuates both peripheral and cerebral inflammation leading to delayed progression of liver disease and HE in mice with ALF due to toxic liver injury. These results suggest that etanercept may provide a novel therapeutic approach for the management of ALF patients awaiting liver transplantation.

## Introduction

Acute liver failure (ALF) is a rare but life-threatening consequence of an abrupt loss of hepatic function in a patient with no previous history of liver disease. ALF may occur as a result of viral infections, liver ischemia, metabolic errors, exposure to drugs or hepatotoxins (acetaminophen, mushroom poisoning) or other unknown causes [1], [2]. Although potentially reversible, it can lead to jaundice, hepatic encephalopathy (HE), coagulopathy, multiorgan failure and ultimately death within days. Mortality rates are high in patients with ALF (≈ 80%) and, in cases where liver regeneration is absent or insufficient to maintain life, liver transplantation remains the only curative treatment option. However, one-third of ALF patients are not eligible for liver transplantation and one-fourth of the patients listed die while waiting for a transplant [3]. These facts underscore the importance of clarifying the pathophysiologic mechanisms of ALF and the urgent need to find therapies capable of delaying the progression of the disease.

[LOOSES]Loss of liver function has detrimental effects on multiple organs, both due to the release of toxic factors from the injured liver and to the loss of key hepatic detoxifying pathways. ALF, in particular, is associated with serious neurological complications, including brain edema and HE, a neuropsychiatric disorder characterized by severe cognitive and psychiatric disturbances ranging from alteration of consciousness to coma [4]. For decades, ammonia has been thought to play a major role in the pathogenesis of the neurological complications of ALF, but recent studies in patients and in animal models strongly suggest that inflammation, acting alone or in concert with ammonia, may also be involved [5].

Inflammation is an important feature of ALF and pro-inflammatory cytokine levels are elevated independently of the etiology of the underlying liver disease [6], [7]. Among the pro-inflammatory cytokines, tumor necrosis factor-alpha (TNF-α) is a potent cytokine that exerts pleiotropic inflammatory and immunological functions by triggering synthesis of downstream targets such as interleukin-6 (IL-6) [8]. Levels of circulating TNF-α are increased in ALF patients and are associated with a poor prognosis [7], [9]. Nevertheless, based on previous studies using TNF-α-lowering strategies, the precise role of TNF-α in toxic liver injury remains controversial. Neutralizing antibodies to TNF-α provide either only partial protection or are ineffective in preventing liver injury in mice administered hepatotoxic doses of acetaminophen and TNF-α knockout mice showed similar sensitivity to acetaminophen compared to wild type mice [10]–[12]. However, progression of HE is significantly delayed in azoxymethane-induced ALF mice lacking the TNF receptor [13].

Etanercept is a dimeric fusion protein consisting of two ligand-binding domains of the soluble human TNF receptor (sTNFR2) linked to the FC fragment of human immunoglobulin G1 (IgG1). It binds to TNF-α and renders it biologically unavailable and thus ineffective. Etanercept is currently used for the treatment of chronic inflammatory diseases such as rheumatoid arthritis and psoriasis [14] and is effective in neutralizing TNF-α in animal models of traumatic brain injury [15], endotoxemia [16] and spinal cord injury [17], leading to improved outcomes.

In order to elucidate the role of TNF-α in toxic liver failure in mice, the protective effects of etanercept on the progression of liver injury and HE were investigated using a well-characterized model of ALF resulting from azoxymethane (AOM) hepatotoxicity. Results of the present study indicate that administration of etanercept delays the progression of liver disease and HE by attenuating the peripheral and central inflammation, which are characteristic of this model of ALF. This suggests that TNF-α plays a key role in toxic liver injury and that etanercept may provide a novel therapeutic approach for the management of ALF patients awaiting liver transplantation.

## Materials and Methods

### Animals

All procedures involving animals were carried out in accordance to the Guidelines of the Canadian Council of Animal Care and protocols were approved by the Animal Research Committee at Saint-Luc Hospital (CHUM). Adult male C57BL6 mice (20–30 g) (Charles River, Saint-Constant, QC, Canada) were maintained under controlled conditions of temperature and humidity, in a 12 h light/dark cycle, and supplied with standard laboratory chow and water *ad libitum*. All animals were free of infection at the onset of the experiment.

### Animal Treatments and Experimental Design

In a first set of experiments, the effects of AOM on the progression of liver dysfunction were studied over time. Mice received a single injection of AOM (100 µg/g; i.p.) (Sigma-Aldrich, St. Louis, CO, USA) dissolved in 100 µl saline as previously described [18]. Control mice received an equivalent volume of saline. Following AOM injection, body temperature was periodically monitored with a rectal probe and rigorously maintained in the range of 36.5–37.5°C using heating pads and lamps. Glycemia was monitored and kept at 5–7 mM by subcutaneous injections of dextrose 10%. Mice were sacrificed by decapitation at 3 h, 6 h, 9 h and 12 h following AOM injection and at coma stages of HE (defined as the loss of corneal reflex, occurs 18–20 h after AOM injection) after anesthesia with a ketamine/xylazine cocktail (50 and 9 mg/kg, respectively; i.p.). Plasma samples were immediately collected from the heart into heparinized tubes, centrifuged (10 min, 10 000 g) and kept at −70°C until use.

In a second set of experiments, the effect of etanercept on the progression of HE (time to coma) was investigated. Mice received a single injection of etanercept (10 mg/kg, i.p.) (Enbrel; Amgen Inc., Thousand Oaks, CA, USA) or saline 30 min prior to, or 3 h or 6 h after administration of AOM (100 µg/g; i.p.). In preliminary experiments, non-specific human immunoglobulin G was chosen as a control vehicle and results showed that it worsens liver damage and precipitates the onset of coma stages (data not shown). Saline was therefore used as a control vehicle rather than human immunoglobulin G. Temperature and glycemia were carefully monitored in all animals as described above until the onset of coma stages of encephalopathy.

In a third set of experiments, the protective effects of etanercept were investigated on liver damage, oxidative stress markers in liver and brain, and microglial activation. Mice received a single injection of etanercept (10 mg/kg; i.p.) or saline 30 min prior to AOM (100 µg/g; i.p.). Temperature and glycemia were carefully monitored in all animals as described above. Etanercept-treated mice and control mice were sacrificed in parallel with saline-treated comatose AOM mice (loss of corneal reflex). Plasma samples were immediately collected from the heart into heparinized tubes, centrifuged (10 min, 10 000 g) and kept at −70°C. Brain (frontal cortex) and liver samples were collected and kept at −70°C until use.

### Histological Assessment of Liver Damage

Livers were fixed overnight by immersion in 10% buffered formalin. Paraffin-embedded specimens were prepared and sections (6 µm) were mounted on Superfrost plus microscope slides (Fisher Scientific, Pittsburg, PA, USA). HPS (hematoxylin-phloxin-saffron) staining was performed according to a standard protocol and liver pathology was assessed by an investigator who was blinded to the experimental treatment groups.

### Biochemical Assays

Plasma samples were diluted 10-fold in saline and alanine aminotransferase (ALT) and aspartate aminotransferase (AST) activities were assayed with an automated analyzer. Ammonia levels in plasma were determined using a commercial ammonia assay kit (Sigma-Aldrich) based on a colorimetric method using the glutamate dehydrogenase enzymatic reaction. The relative standard deviation of the kit was 1–2%. Samples were diluted 40-fold in assay diluent and absorbance was read at 340 nm.

### Determination of Reduced and Oxidized Glutathione

Total (GSH_t_) and reduced (GSSG) glutathione were analyzed in liver and brain (cerebral cortex) using a colorimetric assay (Oxis International, Foster City, CA, USA). Briefly, tissue samples (25 mg) were homogenized in 10 volumes of ice cold 5% metaphosphoric acid. For GSSG determination, a volume of the thiol-scavenging reagent 1-methyl-2-vinylpyridinium trifluoromethanesulfonate (M2VP) was quickly added to 4 volumes of homogenates. All samples (GSHt and GSSG) were centrifuged (10 000 g, 15 min at 4°C). Supernatants were then diluted with assay buffer, loaded onto a microplate and mixed with equal volumes (50 µl) of DTNB chromogen (5,5′-dithiobis-2-nitrobenzoic acid), glutathione reductase and NADPH according to the manufacturer’s protocol. Absorbance was monitored at 412 nm for 10 min. Total and oxidized glutathione were determined from standard curves. Results were expressed in µmoles per g wet weight. The GSH/GSSG ratio was calculated as (GSH_t_ –2GSSG)/GSSG.

### IL-6 Assays in Plasma and Brain

IL-6 levels were measured in plasma and brain using an enzyme-linked immunosorbent assay (ELISA) kit specific for mouse IL-6 (eBioscience, San Diego, CA, USA). Detection limit was 4 pg/mL. For plasma IL-6 measurements, samples were diluted 15-fold in assay diluent and incubated overnight at 4°C. For IL-6 measurements in brain, mice were transcardially perfused with saline and brains rapidly removed, flash frozen in liquid nitrogen and kept at −70°C. Cerebral cortex was homogenized in 50 mM Tris, 1 mM EDTA (pH 7.5) containing a protein inhibitor cocktail (Sigma-Aldrich) and centrifuged at 12 000 g for 45 min. Protein concentrations were measured using a DC protein assay kit (Bio-Rad Laboratories, Hercules, CA, USA). Cytosolic fractions were then diluted 5-fold in assay diluent and incubated overnight at 4°C. The plates were read at 450 nm and values at 570 nm were subtracted. Absorbance was converted to pg/ml using a standard curve prepared with recombinant mouse IL-6. For cerebral IL-6 measurements, levels were expressed in pg/mg of proteins.

### TNF-α and CD40L Measurements in Plasma

Plasma TNF-α and CD40L were measured using specific ELISA kits (eBioscience) according to the manufacturer’s protocol. Detection limits were 8 pg/ml for TNF-α and 0.14 ng/ml for CD40L. For TNF-α measurement, plasma samples were diluted 3-fold in assay diluent and incubated overnight at 4°C. The plate was read at 450 nm and values at 570 nm were subtracted. Absorbance was converted to pg/ml using a standard curve prepared with recombinant mouse TNF-α. For CD40L measurement, plasma was diluted 4-fold in assay diluent and incubated 2 h at room temperature. The plate was read at 450 nm and absorbance was then converted to pg/ml using a standard curve prepared with recombinant mouse CD40L.

### Western Blot Analysis

Brain samples (frontal cortex) from AOM mice treated with etanercept were homogenized in ice-cold buffer (50 mM Tris–HCl, 1 mM EDTA, pH 7.6) containing a protease inhibitor cocktail (Sigma-Aldrich) and centrifuged at 12 000 g for 45 min. Protein concentrations were measured using a DC Bio-Rad protein assay kit. Proteins (120 µg) were solubilized in Laemmli buffer (50 mM Tris–HCl, pH 6.8, 10% glycerol; 2% sodium dodecyl sulfate (SDS), 10% dithiothreitol, 0.1% bromophenol blue) and boiled for 5 min. Proteins were resolved on 10% denaturing SDS-polyacrylamide gels and transferred overnight to polyvinylidene fluoride membranes (Bio-Rad Laboratories). Ponceau S staining was used to verify equal loading. The membranes were blocked for 1 h in Tris-buffered saline (TBS) containing 5% dry milk and 0.1% Tween 20, then incubated for 1 h with anti-human (Fc specific) IgG antibody coupled to horseradish peroxidase (Sigma-Aldrich; 1/25 000). After washing with TBS, peroxidase activity was detected by chemiluminescence using the ECL detection system (Amersham, Arlington Heights, IL, USA). The lower limit of detection of etanercept (3.5 ng) was determined by mixing serial dilutions of etanercept with 120 µg of total protein extracted from frontal cortex of etanercept-treated mice (data not shown).

### Immunohistochemistry

Mice were deeply anaesthetized with ketamine and xylazine and perfused transcardially with 100 ml ice-cold saline followed by 100 ml formalin (containing 4% paraformaldehyde in phosphate buffered saline (PBS), pH 7.0). Brains were removed, post-fixed in 4% formalin at 4°C for 12 h and transferred into ice-cold PBS (137 mM NaCl, 10 mM Na_2_HPO_4_, 2.7 mM KCl, 1.8 mM, KH_2_PO_4_; pH = 7.4) containing 0.09% sodium azide solution for storage. Free-floating 50 µm thick coronal sections were obtained using a vibratome and mounted on Superfrost Plus slides. Sections were incubated with 0.3% hydrogen peroxide in methanol for 15 min to block endogenous peroxidase activity and with 10% rabbit serum and 0.2% Triton X-100 to block non-specific binding sites. Sections were then incubated at 4°C overnight with rat anti-mouse CD11b (OX-42) (1/100) (AbD Serotec, Raleigh, NC, USA). After washing with PBS, sections were incubated for 1 h with rabbit anti-rat biotinylated secondary antibody (1/200) (Vector Laboratories, Burlingame, CA, USA) and thereafter with Vectastain ABC reagent (Vector Laboratories). OX-42 immunoreactivity was detected by incubation with 3–3′-diaminobenzidine containing urea-hydrogen peroxide (Sigma-Aldrich). Sections were dehydrated stepwise in ethanol and xylene and mounted with Permount (Fisher Scientific). Sections in which the primary antibody was omitted were used as negative controls and showed no immunoreactivity.

### Statistical Analysis

All data are expressed as the mean ± SEM and statistical analysis was performed using one-way analysis of variance (ANOVA) followed by Tukey’s post hoc analysis. A P value of less than 0.05 was considered to indicate a significant difference. Data were analyzed by using Prism 5.0 software (GraphPad Prism 5.0, San Diego, CA, USA).

## Results

### Circulating TNF-α and IL-6 Increase before the Onset of Hepatic Damage Following AOM Administration

Levels of circulating TNF-α and IL-6 over time were first evaluated following AOM administration. Plasma TNF-α levels were significantly increased 9 h after AOM injection (12.7-fold; p<0.001) and remained at similar levels 12 h after AOM injection and at coma stages of encephalopathy (Table 1). Similarly, IL-6 levels were significantly increased 9 h following AOM treatment compared to control mice (691-fold; p<0.001) and remained elevated until the onset of coma (Table 1). On the other hand, transaminase activities and plasma ammonia levels tended to increase during the progression of liver disease and reached statistical significance by the onset of coma. Compared to saline-treated controls, AST activity was increased by 17.6-fold (p<0.001), ALT activity was increased by 88.8-fold (p<0.001), and plasma ammonia levels were increased 6.7-fold (p<0.001) at coma stages of encephalopathy (Table 1).

**Table 1:** Effect of azoxymethane (AOM) on plasma transaminase activities, ammonia, TNF-α and IL-6 levels over time.

	Control	3 h	6 h	9 h	12 h	Coma
AST (U/L)	176.7±31.8	275.0±65.1	303.3±65.7	792.5±207.1	1488±736.6	3113±380.3*
ALT (U/L)	53.3±3.3	57.5±4.8	104.0±24.3	120.0±12.2	312.8±96.2	4733±676.3*
Ammonia (µM)	86.7±15.8	88.9±19.1	133.3±28.4	223.9±10.4	223.9±20.1	577.8±93.0*
TNF-α (pg/mL)	10.8±0.4	21.0±1.5	46.2±2.9	137.1±2.5*	143.7±11.09*	120.4±15.5*
IL-6 (pg/mL)	12.4±4.3	65.4±10.6	803.7±129.5	8568±1174*	6213±1266*	10609±653.1*

Mice (n = 5) were sacrificed at various time points indicated following AOM injection.

* p<0.001 vs. control by ANOVA.

### Etanercept Delays the Progression of Hepatic Encephalopathy

**Figure 1:**
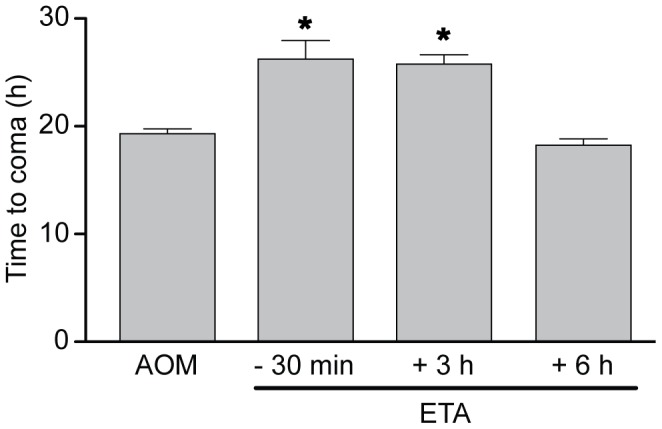
Etanercept significantly delays the progression of hepatic encephalopathy in mice with AOM-induced ALF. Time to coma (loss of corneal reflex) in ALF mice treated with etanercept (ETA) 30 min prior to, or 3 h and 6 h after AOM treatment. Data represent mean ± SEM of n = 6 animals in each group. *p<0.001 vs. Saline.

The effects of etanercept administration on the progression of encephalopathy were next investigated. Following AOM administration, saline-treated mice as well as etanercept-treated mice developed HE progressing from lethargy and ataxia to loss of righting and corneal reflexes (coma stage). Saline-treated mice became comatose 19.3±0.4 h following AOM injection whereas the onset of coma was significantly delayed in mice pretreated (−30 min) with etanercept (26.2±1.7 h; p<0.001) or injected with etanercept 3 h after AOM (25.8±0.9 h; p<0.001). The progression of encephalopathy in mice injected with etanercept 6 h after AOM treatment was not significantly different from saline-treated AOM mice (Fig. 1).

### Etanercept Treatment Attenuates Hepatocellular Damage and Normalizes Plasma Ammonia Levels

**Figure 2:**
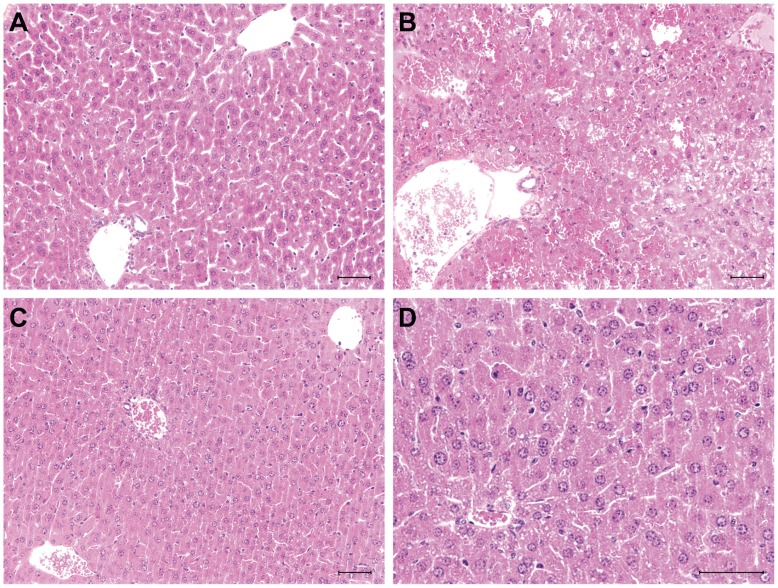
Etanercept attenuates liver damage in mice with AOM-induced ALF. Etanercept was administered 30 min before AOM. Representative liver sections from (A) saline-treated control mice, (B) AOM-induced ALF comatose mice and (C and D) etanercept-treated ALF mice. ALF mice show areas of confluent hemorrhagic necrosis, microvesicular steatosis as well as sinusoidal dilatation and congestion. Note the presence of minor microvesicular steatosis with little necrosis and sinusoidal congestion following etanercept treatment. Representative pictures from n = 6 mice are shown. Scale bar : 50 µm.

AOM induced microvesicular steatosis with extensive degrees of necrosis and hemorrhagic congestion predominantly affecting midzonal and centrilobular regions, as previously reported [18] (Fig. 2). Etanercept pretreatment (−30 min) significantly attenuated hepatic necrosis and hemorrhagic congestion with concomitant decreases in AST (3.4-fold; p<0.001) and ALT (9.5-fold; p<0.001) plasma levels compared to saline-treated ALF mice. Etanercept treatment also reduced blood ammonia levels 3.9-fold (p<0.001) to values comparable to those observed in saline-treated mice (Table 2). Interestingly, etanercept pretreatment (−30 min) was also protective in mice with acetaminophen-induced liver damage as evidenced by improved liver histopathology, and significantly reduced levels of transaminases and ammonia (data not shown).

**Table 2:** Effects of etanercept on plasma transaminase activities and ammonia levels.

	Control	AOM-induced ALF	
		Vehicle-treated	Etanercept-treated
AST (U/L)	112.9±8.0	3674±306.5*	1093±190.1†
ALT (U/L)	64.5±3.9	3699±397.6*	390.0±74.8†
Ammonia (µM)	101.4±5.3	500.4±70.4*	129.5±12.0†

Mice were administered etanercept 30 min prior to azoxymethane (AOM) or saline (Vehicle). Control mice received only saline. Data represent mean ± SEM of n = 12 in each group.

* p<0.001 vs. Control;

† p<0.001 vs. vehicle-treated.

### Etanercept Normalizes Plasma TNF-α, IL-6 and CD40L Levels

**Figure 3:**
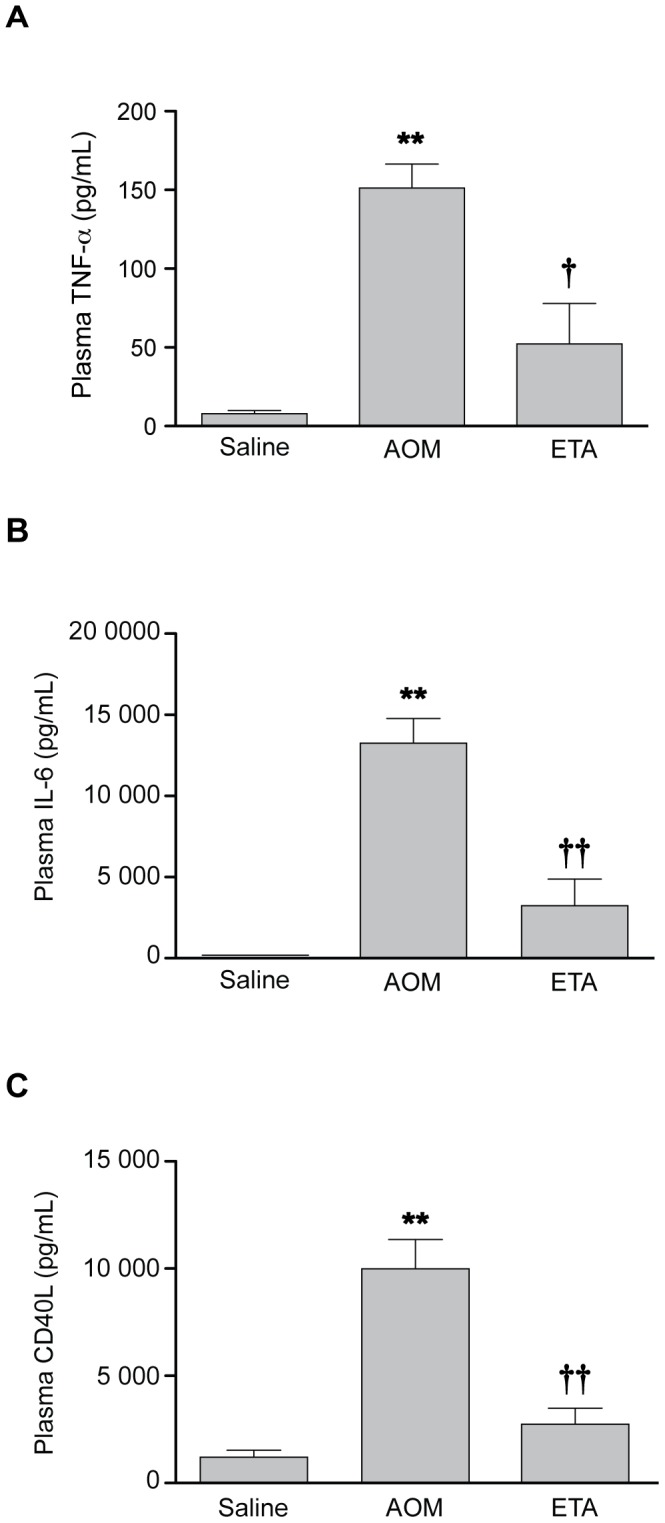
Etanercept treatment significantly attenuates plasma TNF-α, IL-6 and CD40L in mice with AOM-induced ALF. Etanercept was administered 30 min before AOM. Plasma TNF-α (A), IL-6 (B) and CD40L (C) were quantified by ELISA. Data represent mean ± SEM of n = 12 animals in each group for IL-6, and n = 6 for TNF-α and CD40L. **p<0.001 vs. Saline; †p<0.05 vs. AOM; ††p<0.001 vs. AOM.

The effect of etanercept on systemic inflammation was investigated by measuring levels of the pro-inflammatory mediators TNF-α, IL-6 and CD40L. In addition to increases of TNF-α, significant increases in plasma IL-6 (732.5-fold; p<0.001) (Fig. 3B) and CD40L (8.3-fold; p<0.001) (Fig. 3C) were observed in AOM-treated mice at coma stages of encephalopathy. Compared to saline-treated AOM mice, etanercept-pretreated mice displayed significantly lower levels of plasma TNF-α (2.5-fold; p<0.05), IL-6 (4.1-fold; p<0.001) and CD40L (3.7-fold; p<0.001).

### Etanercept Attenuates Cerebral IL-6 Levels and Microglial Activation

**Figure 4:**
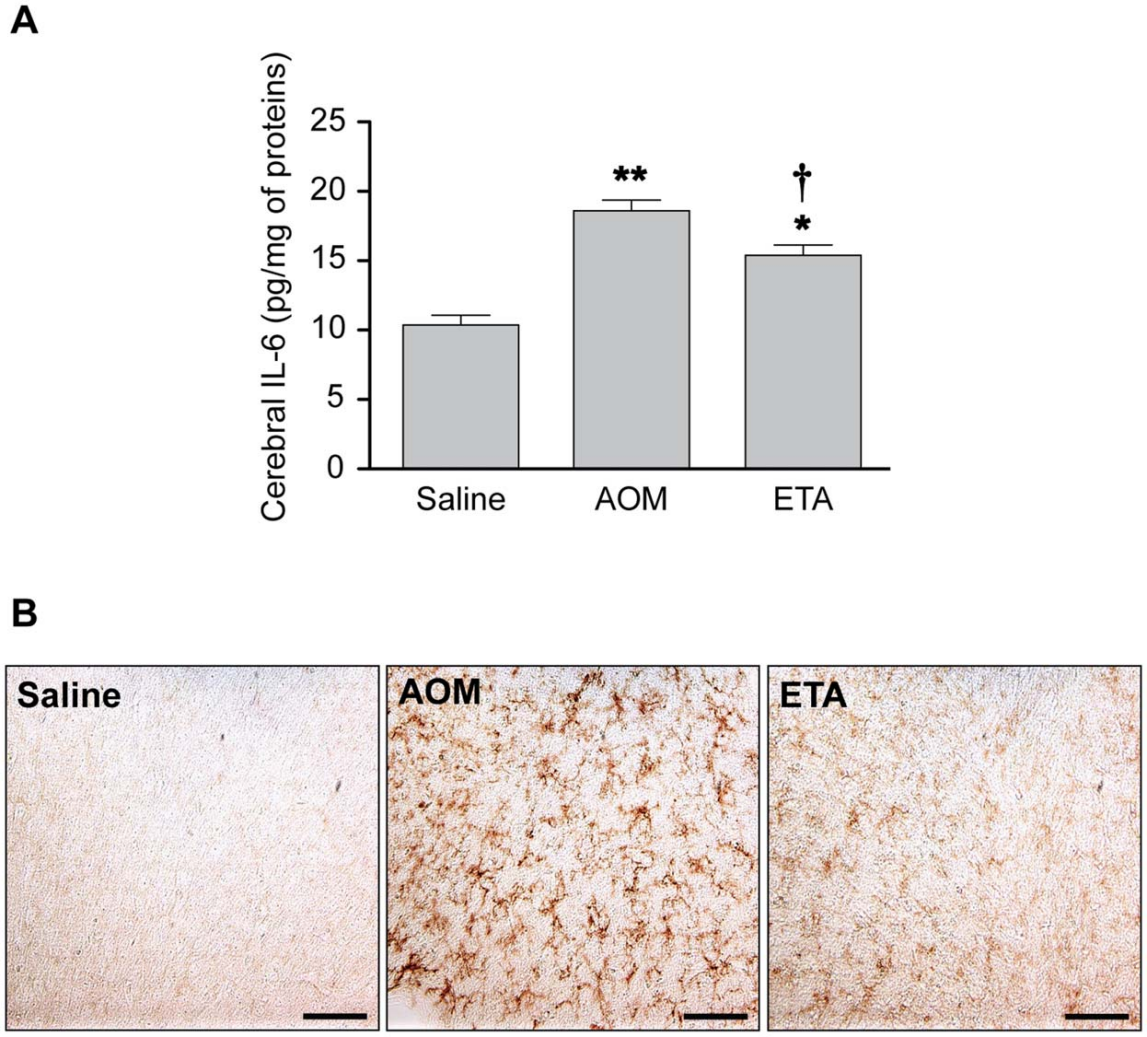
Etanercept treatment attenuates IL-6 levels and microglial activation in the brain of mice with ALF. Etanercept was administered 30 min before AOM. (A) Brain IL-6 levels were measured in cytosolic fractions of cerebral cortex by ELISA. Data represent mean ± SEM of n = 6 animals in each group. *p<0.01 vs. Saline; **p<0.001 vs. Saline; †p<0.05 vs. AOM. (B) Representative micrographs showing the effect of ALF on OX-42 (CD11b) staining in cerebral cortex from saline-treated control, AOM-treated mice at coma stages of encephalopathy, and etanercept-treated ALF mice. Scale bar : 100 µm.

Administration of AOM led to neuroinflammation as evidenced by a significant increase of brain IL-6 levels (1.8-fold; p<0.001) compared to saline-treated controls, and treatment with etanercept significantly attenuated brain IL-6 levels induced by AOM (1.2-fold; p<0.05) (Fig. 4A). In addition, OX-42 immunoreactivity (CD11b), a marker of activated microglia/macrophages, was significantly increased in the cerebral cortex of AOM mice at coma stages of encephalopathy whereas no immunoreactivity was detected in saline treated-controls (Fig. 4B). OX-42 immunoreactivity was significantly attenuated in AOM mice pretreated with etanercept.

**Figure 5:**
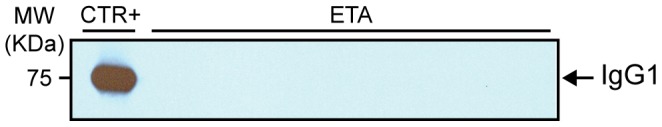
Western blot analysis of human IgG in the cerebral cortex of etanercept-treated AOM mice.

To assess whether the attenuating effects of etanercept on neuroinflammation were due to a local action following its entry into the brain or secondary to its peripheral effects, western blot analysis was performed to determine whether etanercept could be detected in the brain parenchyma following its administration in AOM mice. Cerebral cortex extracts from perfused etanercept-treated AOM mice did not show detectable levels of etanercept (Fig. 5).

### Etanercept Treatment Attenuates Oxidative Stress in Liver and Brain

**Figure 6:**
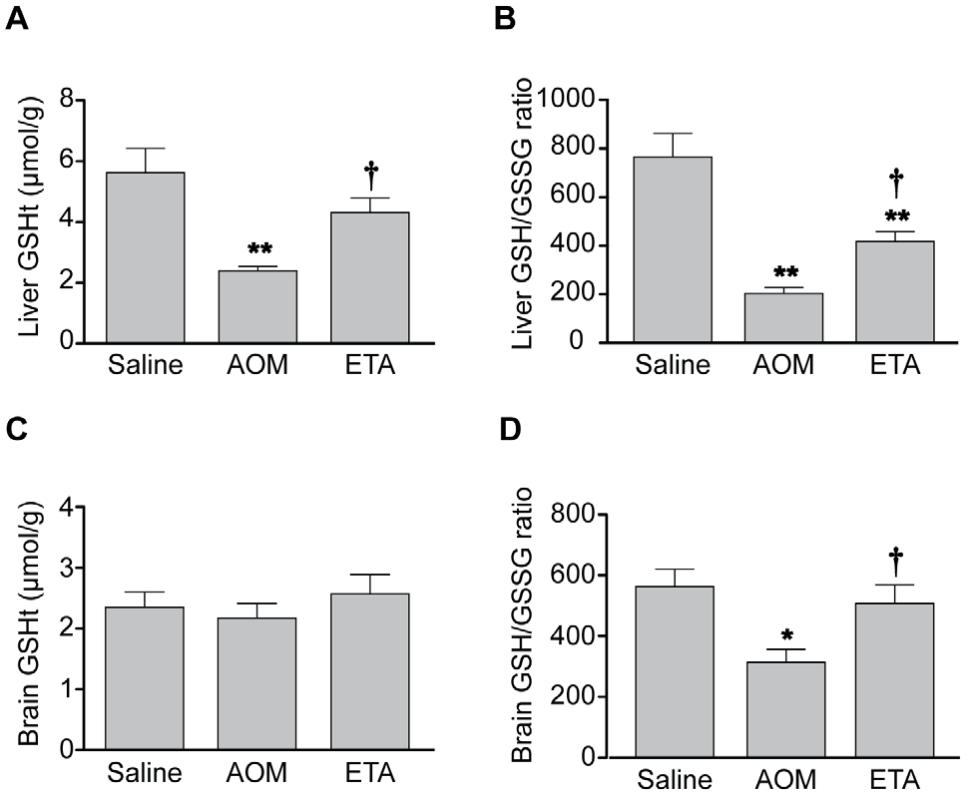
Etanercept treatment attenuates oxidative stress in liver and brain. Etanercept was administered 30 min before AOM. Total glutathione (GSH_t_) and GSH/GSSG ratio were determined in liver (A and B, respectively) and brain (C and D, respectively). Data represent mean ± SEM of n = 6 (liver), n = 12 (brain) and are expressed as µmol/g wet weight (A and C). *p<0.05 vs. Saline; **p<0.001 vs. Saline; †p<0.05 vs. AOM.

The protective effect of etanercept on oxidative stress, which is frequently associated with inflammation, was next investigated. At coma stages of encephalopathy, total hepatic glutathione stores (GSH_t_) were significantly depleted in AOM-treated mice compared to saline-treated controls (2.3-fold; p<0.001) and the reduced/oxidized glutathione ratio (GSH/GSSG) was significantly decreased (3.75-fold; p<0.001) (Fig. 6A and 6B). Etanercept treatment prevented the depletion of total hepatic glutathione content (1.8-fold; p<0.05) and significantly increased the GSH/GSSG ratio (2-fold; p<0.05). In brain, total glutathione levels of AOM mice at coma stages of encephalopathy were similar to those of saline-treated controls (Fig. 6C). However, the GSH/GSSG ratio was decreased in brains of AOM mice compared to saline-treated controls (1.8-fold; p<0.05) and significantly increased by etanercept treatment to values similar to those observed in saline-treated mice (1.6-fold; p<0.05) (Fig. 6D).

## Discussion

Findings of the present study indicate that TNF-α levels are increased early during AOM-induced ALF and that etanercept administration significantly delays the progression of HE by reducing hepatocellular damage, decreasing both systemic and central inflammation as well as hepatic and cerebral oxidative stress in these animals. These results add further support to the notion that TNF-α plays a major role in the pathogenesis of ALF.

TNF-α is a key cytokine that exerts pleiotropic effects ranging from proliferative responses, inflammatory effects and modulation of immune responses, to destructive cellular outcomes such as apoptotic and necrotic cell death. TNF-α is produced as a transmembrane (tmTNF-α) or a membrane-cleaved circulating cytokine (sTNF-α) by various cell types such as macrophages, lymphocytes, cerebral microglia and astrocytes, and hepatic Kupffer cells. It has been suggested that TNF-α plays a major role in the pathogenesis of HE associated with ALF where the circulating levels of this cytokine are significantly increased both in patients and animal models [19]. Moreover, a direct correlation has been established between TNF-α levels, severity of ALF and prognosis [7], [9]. In the present study, plasma TNF-α levels reached statistical significance 9 h after AOM administration and clearly preceded the onset of coma and massive hepatocyte cell death which occurred only at coma stages of HE, as shown by large increases in ammonia and transaminase levels. These observations suggest that TNF-α plays a role early in the pathogenesis of AOM-induced ALF and HE. These findings are in accordance with other experimental models of ALF where elevated serum levels of TNF-α have been observed shortly after the initiating insult. For example, plasma TNF-α levels were increased as early as 4 h following injection of a single dose of acetaminophen in mice and were correlated with the onset of hepatic damage [20].

TNF-α performs its biological functions as a homotrimer that binds to p55 and p75 TNF receptors (TNFR 1 and 2, respectively) and induces a variety of intracellular pathways regulating the transcription of a large number of genes implicated in host defence [21]. Importantly, TNF-α triggers within hours the secretion of multiple pro-inflammatory mediators, among them IL-6 [8]. Results of the present study demonstrated that plasma IL-6 levels were significantly increased 9 h after AOM-induced hepatotoxicity and correlated with plasma TNF-α levels, which underscores the rapid pro-inflammatory effect of TNF-α and confirms the presence of systemic inflammation in the AOM model of ALF.

Systemic administration of etanercept has been shown to be effective in neutralizing TNF-α in various animal models of central nervous system injury, thus leading to improved outcomes [15], [17]. Etanercept is a fusion protein of two TNFR2 extracellular domains linked to the Fc fragment of human immunoglobulin 1 (IgG1). It binds to a single sTNF-α trimer or tmTNF-α in a 1∶1 ratio thereby acting as a competitive inhibitor of TNF-α [22], [23]. Etanercept has a 50-fold greater affinity and is 1000-fold more efficient than the endogenous monomeric sTNF-α receptor as assessed *in vitro* by inhibition of TNF-α binding or bioactivity [16]. The half-life of etanercept *in vivo* is three days, which is five times that of monomeric sTNF-α receptor [24]. These characteristics, as well as the high volume of distribution of etanercept, result in a greater ability to neutralize the biologic effects of TNF-α, suggesting that it may be a suitable anti-TNF-α therapy in our model of ALF. Results of the present study show that systemic administration of etanercept 30 min before or 3 h after AOM leads to delayed progression of HE and onset of coma as well as improved hepatic function. The protective effect of etanercept on AOM-induced liver damage was evidenced by reduced hepatocellular necrosis, microvesicular steatosis and hemorrhagic congestion, and by a preventive effect on the increases in plasma levels of ammonia and transaminases. Similar protective effects of etanercept were also observed in acetaminphen-induced liver injury, suggesting that the beneficial effects of etanercept are not specific to AOM-induced liver damage and that this drug may be useful in liver damage from various aetiologies.

Innate and adaptive immune responses occur during acute liver injury and are accompanied by the secretion of large amounts of pro-inflammatory cytokines [25], [26]. In the present study, we focused on plasma IL-6 and CD40L, two pro-inflammatory mediators implicated in the inflammatory cascade mediated by TNF-α, in order to evaluate the biological effects of TNF-α inhibition. Etanercept administration prevented the increases of IL-6 and CD40L in mice with AOM-induced ALF confirming that the protective effects of etanercept are the consequence of the peripheral inhibition of the biological action of TNF-α. Importantly, previous studies have demonstrated the implication of these cytokines in the pathogenesis of ALF. Indeed, plasma IL-6 levels are significantly increased in patients with fulminant hepatitis and are indicative of a poor prognosis [27]. On the other hand, the dyad of the TNF family members CD40/CD40L is implicated in the inflammatory cascade that leads to ALF and induces the synthesis of pro-inflammatory cytokines such as TNF-α and IL-6 [28]. In liver, increased expression of CD40L and its receptor CD40 constitute an early mechanism for liver cell damage in both human and murine fulminant hepatic failure [29] and a recent study has demonstrated that overexpression of hepatic CD40L in mice induces ALF [30]. Moreover, serum levels of CD40L are higher in patients with fulminant hepatitis than in patients with acute hepatitis or controls and are associated with a poor prognosis [31].

Interestingly, administration of etanercept 6 h after AOM treatment had no effect on the progression of HE, suggesting that the inflammatory cascade driven by TNF-α might already be too extensive at this stage to be reversed by etanercept. Indeed, although only a slight and non-significant increase in plasma TNF-α is observed 6 h after AOM administration, it is possible that the activation of Kupffer cells and the secondary recruitment of immune cells such as monocytes and neutrophils may already have led to an irreversible inflammatory response locally, accompanied by hepatocellular damage [32].

Oxidative stress is a component of the innate inflammatory response that develops as a consequence of injury. Our unit has previously demonstrated that oxidative stress plays a key role in the physiopathology of ALF in mice resulting from AOM hepatotoxicity, and that the protective effects of mild hypothermia or N-acetylcysteine in this model is mediated by an attenuation of plasma levels of pro-inflammatory cytokines, and a reduction of both the depletion of liver glutathione stores and of the GSH/GSSG ratio [33], [34]. Results of the present study also demonstrate that systemic sequestration of TNF-α by etanercept attenuates the oxidative stress response associated with inflammatory pathways by preventing the depletion of liver glutathione stores and attenuating the decrease in hepatic GSH/GSSG ratio. Maintenance of hepatic glutathione stores thus appears to be an important mechanism by which peripheral inhibition of TNF-α prevents further hepatocellular damage.

Acute liver failure is frequently associated with serious neurological complications including HE and cerebral herniation. For decades, a great deal of attention has been focused on ammonia as the main agent responsible for the central nervous system complications of ALF. However, ammonia-lowering strategies are of limited value in preventing the cerebral complications of ALF [35], and recent studies strongly suggest that inflammation acting alone or in concert with ammonia plays an important role in the pathogenesis of HE [5], [37]. Results of the present study demonstrated that systemic administration of etanercept also attenuated the neuroinflammatory response associated with AOM-induced ALF, as shown by lower cerebral IL-6 levels, attenuated microglial activation and a preserved GSH/GSSG ratio. It is important to note that OX-42 is a marker of both activated microglia and macrophages, therefore it cannot be excluded that OX-42 positive cells may in part result from monocyte infiltration as shown in another model of liver damage [38]. Moreover, analysis of cerebral cortex extracts from etanercept-treated ALF mice failed to show the presence of significant amount of etanercept in the brain, suggesting that the 150 kDa recombinant complex does not cross the blood-brain barrier (BBB) following AOM administration and that BBB integrity was maintained. These results suggest that the beneficial effects of etanercept on HE in AOM-treated ALF mice are primarily mediated peripherally *via* an improvement of liver function. However, it cannot be excluded that etanercept is also acting indirectly by blocking the transduction of pro-inflammatory signalling to the brain. Immune-to-brain signalling can indeed occur *via* mechanisms such as i) entry of cytokines through brain regions lacking a functional BBB; ii) active cytokine transport at the BBB; iii) activation of brain endothelial cells resulting in release of second messengers into the brain; iv) signalling through afferent nerve fibres in the periphery; v) recruitment of activated immune cells into the brain parenchyma [39]. Moreover, there is evidence that systemic pro-inflammatory cytokines such as IL-6 and TNF-α increase the permeability of cerebrovascular endothelial cells to ammonia [40] suggesting that beneficial effects of etanercept on neuroinflammation during ALF may also result from the prevention of an increase of BBB permeability to ammonia.

In conclusion, the present study demonstrates that TNF-α plays a key role in the pathogenesis of ALF and HE following AOM-induced hepatotoxicity in the mouse. Systemically administrated etanercept delayed the onset and progression of HE by attenuating systemic inflammation and its deleterious consequences i.e hepatocellular damage, oxidative stress and neuroinflammation. These results suggest that etanercept has the potential to provide a promising therapeutic approach for the management of ALF in patients awaiting liver transplantation.
